# PeanutDB: an integrated bioinformatics web portal for *Arachis hypogaea* transcriptomics

**DOI:** 10.1186/1471-2229-12-94

**Published:** 2012-06-19

**Authors:** Xiaohong Duan, Emily Schmidt, Pei Li, Douglas Lenox, Lin Liu, Changlong Shu, Jie Zhang, Chun Liang

**Affiliations:** 1 State Key Laboratory for Biology of Plant Diseases and Insect Pests, Institute of Plant Protection, Chinese Academy of Agricultural Sciences, Beijing, 100193, China; 2 Department of Botany, Miami University, Oxford, OH, 45056, USA; 3 Department of Computer Science and Software Engineering, Miami University, Oxford, OH, 45056, USA

**Keywords:** Peanut, *Arachis hypogaea*, Transcriptome sequencing, Transcriptome assembly, Database, PeanutDB, SNP, SSR, Functional annotation

## Abstract

**Background:**

The peanut (*Arachis hypogaea*) is an important crop cultivated worldwide for oil production and food sources. Its complex genetic architecture (*e.g.*, the large and tetraploid genome possibly due to unique cross of wild diploid relatives and subsequent chromosome duplication: 2n = 4x = 40, AABB, 2800 Mb) presents a major challenge for its genome sequencing and makes it a less-studied crop. Without a doubt, transcriptome sequencing is the most effective way to harness the genome structure and gene expression dynamics of this non-model species that has a limited genomic resource.

**Description:**

With the development of next generation sequencing technologies such as 454 pyro-sequencing and Illumina sequencing by synthesis, the transcriptomics data of peanut is rapidly accumulated in both the public databases and private sectors. Integrating 187,636 Sanger reads (103,685,419 bases), 1,165,168 Roche 454 reads (333,862,593 bases) and 57,135,995 Illumina reads (4,073,740,115 bases), we generated the first release of our peanut transcriptome assembly that contains 32,619 contigs. We provided EC, KEGG and GO functional annotations to these contigs and detected SSRs, SNPs and other genetic polymorphisms for each contig. Based on both open-source and our in-house tools, PeanutDB presents many seamlessly integrated web interfaces that allow users to search, filter, navigate and visualize easily the whole transcript assembly, its annotations and detected polymorphisms and simple sequence repeats. For each contig, sequence alignment is presented in both bird’s-eye view and nucleotide level resolution, with colorfully highlighted regions of mismatches, indels and repeats that facilitate close examination of assembly quality, genetic polymorphisms, sequence repeats and/or sequencing errors.

**Conclusion:**

As a public genomic database that integrates peanut transcriptome data from different sources, PeanutDB (http://bioinfolab.muohio.edu/txid3818v1) provides the Peanut research community with an easy-to-use web portal that will definitely facilitate genomics research and molecular breeding in this less-studied crop.

## Background

The peanut (*Arachis hypogaea*), an annual herbaceous legume plant, is an important crop cultivated worldwide for oil production and food sources [1,2]. This cultivated species has a large and complex tetraploid genome (2n = 4x = 40, AABB, 2800 Mb), presenting a serious challenge for its whole genome sequencing [3,4]. It is likely that the cultivated peanut derived from crossing of wild diploid species and underwent subsequent genome duplication [4,5]. Blockage of gene flow from wild diploid to cultivated tetraploid apparently contributes to the low genetic variation in peanut and presents a bottleneck to its genetic improvement [4]. The large and complex genome structures of peanut with low genetic variation apparently make this non-model species remain among the less-studied crops.

As in July 15, 2011, there were a total of 198,156 nucleotide sequences for *Arachis hypogaea* in NCBI GenBank (http://www.ncbi.nlm.nih.gov/nuccore?term=txid3818), including 39,852 Nucleotide (Core Nucleotide Sequences), 150,177 ESTs (Expressed Sequence Tags), and 8,125 GSSs (Genome Survey Sequences). The 150,177 ESTs were deposited by many researchers [3,6-10], and the sampling tissues were mainly seeds (57%), roots (23%) and leaves (19%) [4]. In the same time, the University of Georgia (UGA) submitted more than 1 million 454 sequence reads into NCBI Short Read Archive (SRA) (http://www.ncbi.nlm.nih.gov/bioproject?term=PRJNA49471) for 17 *Arachis hypogaea* L. genotypes. Meanwhile, Illumina transcriptome sequencing of *Arachis hypogaea* L. has been conducted by Institute of Plant Protection, Chinese Academy of Agricultural Sciences and generated about 70 million reads.

As a public genomic database that integrates transcript data from Sanger, 454 and Illumina, PeanutDB currently focuses on the transcriptome analysis of *Arachis hypogaea*. Based on all aforementioned cDNA and mRNA data, we have created our first release of peanut transcriptome assembly consisting of 32,619 contigs. We not only provided Gene Ontology (GO), Enzyme Commission (EC) and Kyoto Encyclopedia of Genes and Genomes (KEGG) annotation to these contigs, but also identified extensively the potential polymorphisms, both single base (*e.g.*, SNPs and single indels) and multiple bases (*e.g.*, mismatches/indels of multiple bases), and simple sequences repeats for each contig. In particular, the whole transcriptome assembly, its functional annotations and relevant SNPs and SSRs are presented in many easy-to-use web views that allow users to search, filter, navigate and visualize their desired data efficiently and easily. Using our in-house tools, sequence alignment for each contig can be displayed in either bird’s-eye view or nucleotide-level resolution, facilitating close examination of assembly quality, genetic polymorphisms, sequence repeats, and sequencing errors. In addition, BLAST + [11] is deployed in PeanutDB so that users can blast their own sequences against our contigs, NCBI peanut datasets, and other datasets if requested. PeanutDB also empowers individual researchers by making all our data downloadable. PeanutDB strives to provide the community an open access web portal that constantly incorporates both open-source and in-house bioinformatics tools. Clearly, our database will be a useful bioinformatics resource that will facilitate genome sequencing and gene annotation and benefit genetic breeding in peanut.

## Construction and content

### Data sources and data processing

The peanut transcriptome data hosted in PeanutDB consists of three different data sources. As in July 15, 2011, we downloaded a total of 198,156 nucleotide sequence reads for *Arachis hypogaea* in NCBI GenBank, including 39,854 Core Nucleotide Sequences and 150,177 Expressed Sequence Tags (dbEST). Among 39,852 Core Nucleotide Sequences, we used 38,425 that are labeled as mRNA. Meanwhile, we obtained a total of 1,040 Million bases of 454 GS FLX reads from NCBI SRA (Short Read Archive) for *Arachis hypogaea*, with the accession numbers of SRX019971, SRX019972, SRX019979, SRX020012 and SRX020014. Simultaneously, 5 Gigabytes of Illumina data were released from the Institute of Plant Protection, Chinese Academy of Agricultural Sciences, which were generated by sequencing the total RNA extractions from leaves, roots and seeds of *Arachis hypogaea* (Genotype: *Baisha1016*). Data cleaning such as adapter removal and low-quality trimming was performed respectively for Illumina reads using CLC Genomics Workbench (CLC bio, version 4.8) and for Sanger and 454 reads using GS *De Novo* Assembler (454 Life Sciences, version 2.6). The final clean sequences used for cDNA clustering contains 187,636 Sanger reads (with a total of 103,685,419 bases), 1,165,168 Roche 454 reads (with a total of 333,862,593 bases), and 57,135,995 Illumina reads data (with a total of 4,073,740,115 bases).

CLC Genomics Workbench was also used to conduct *de novo* transcriptome assembly for clean sequences. Using more stringent parameters (*e.g.*, Length fraction = 0.8, Similarity = 0.9) in CLC Genomics Workbench 4.8, we obtained a total of 32,619 contigs in FASTA format and the relevant SAM file [12] that contains sequence alignment information for all the contigs and their component sequence reads. Phobos [13] was used to identify both perfect and imperfect short sequence repeats (SSRs) with the repeat unit size of 1–12 *nt*. The minimum repeat number was set to be 8 for homopolymers, 5 for dimers, 4 for triplets, and 3 for SSRs with a unit size of 4–12 *nt*. Based on the aforementioned SAM file, our in-house C++ program was used to identify SNPs, single indels, and other mismatches/indels of ≥1 *nt* in size and generate image files for sequence alignment visualization. Along each contig, we identified all putative polymorphic positions, which must be supported (or covered) by at least 10 individual sequence reads and the accumulative occurrence of any polymorphic type (*e.g.*, SNPs, single indels, and other mismatches/indels of ≥1 *nt* in size) is more than 5. For a given putative polymorphic position within a contig, we then determined the occurrence and type of a valid SNP, single indel, and multiple-base mismatch/indel, as long as its occurrence is at least 2 (*i.e.*, there are at least 2 individual sequence reads that have the same of polymorphism type in a given polymorphic position along the contig). Annot8r [14] was used to generate GO, EC and KEGG annotation for every contig. Finally, the processed data including contig annotation, simple sequence repeats, and polymorphisms were dumped into a local MySQL database that can be accessed by various web interfaces (see **Web Interfaces** in **Utility** section). All web interfaces for PeanutDB were developed using PHP and JavaScript.

### Database design and schema

The database design for PeanutDB was intended to be simple and efficient for fast data retrieval needed by our web interfaces. Because we adopted annot8r [14] to provide GO, EC and KEGG annotation to each contig, its relational database design and schema for storing annotation references and hit results are consequently utilized in PeanutDB. In addition, we designed 5 more tables, each of which corresponds to the relevant web interface or view (see below). The table *ContigView* contains summary information for each contig, including the contig length, the total number of sequence reads, sequencing coverage calculated by *(read number × average read length)/(contig length)*, the total number of SNPs and SSRs, and among others. The table *SSRView* stores start and end position, repeat unit size and sequence, repeat number and identity (perfect vs. imperfect SSR) of all SSRs detected in each contig. The table *PolymorphismPosition* hosts information about the putative polymorphic positions along each contig. For example, the position 391 in contig t3818.cdna.v1.contig18450 is polymorphic because 16 sequence reads have T, 502 reads have C, 371 reads have a deletion, and 1 read has G in this position whereas the contig base is C (http://bioinfolab.muohio.edu/txid3818v1/CBrowse/interface/ngctgbrowser.php?ctgname=t3818.cdna.v1.contig18450&pos=391&zoom=2). For each putative polymorphic position along a contig, if there is a point mutation (*e.g.*, one-base mismatch, insertion or deletion), the detailed information will be stored in the table *PolymorphismSNP*; if there is a mismatch, insertion or deletion of more than one base, the detailed information will be stored in the table *PolymorphismOther*.

### Major characteristics of the transcriptome assembly

Table 1 summarizes the major characteristics of our transcript assembly. As shown in Table 1, the contig length for all 32,619 contigs ranges from 91 to 29,281 *nt*, with the N50 of 1,167 *nt*. Among them, 99.1% (*i.e.*, 32,319 out of 32,619) has a length of ≥ 500 *nt*, 37.5% (*i.e.*, 12,225 out of 32,619) has a length of ≥ 1,000 *nt*, and 0.1% (*i.e.*, 44 out of 32,619) has a length of ≥ 5,000 *nt*. The maximum sequence reads for single contig is 505,778, with the coverage of 62,678. For functional annotation, 59.9% contigs (*i.e.*, 19,592 out of 32,619) has valid GO annotation, 23.7% (*i.e.*, 7,744 out of 32,619) has valid EC annotation, and 29.4% (*i.e.*, 9,604 out of 32,619) has valid KEGG annotation (see Table 1). For polymorphism, we have detected a total of 448,598 putative polymorphic positions along all contigs, which means approximate 14 per contig. Among these polymorphic positions, we identified 1,076,427 putative SNPs and single indels >(*i.e.*, 33 per contig) and 64,860 (*i.e.*, 2 per contig) mismatches/indels of ≥ 1 *nt* in size. For simple sequence repeat, we have found 812 different kinds of SSR (*e.g.*, AAG, AG, ATC, AAT are among the most frequently occurred SSRs) and a total of 30,195 putative SSRs for all contigs, which means approximate 1 per contig.

**Table 1:** **Major characters of the peanutDB transcriptome assembly**^**1**^

**Contigs**	**Number**	**%**^**2**^	**Length (*****nt*****)**
The Total Contigs in the Assembly	32,619	100.0	
The Longest Contig			29,281
The Shortest Contig			91
N50 Length^3^			1,167
Contigs with a length > = 500 *nt*	32,319	99.1	
Contigs with a length > = 1,000 *nt*	12,225	37.5	
Contigs with a length > = 5,000 *nt*	44	0.1	
Contigs with valid GO annotation	19,529	59.9	
Contigs with valid EC annotation	7,744	23.7	
Contigs with valid KEGG annotation	9,604	29.4	

^1^ PeanutDB Transcriptome Assembly release: PeanutDB Version 1.0, Apr-18-2012.

^2^ The percentage is calculated using the total contig number (*i.e.*, 32,619) in the assembly as the denominator.

^3^ N50 Length is a statistical indicator of average contig length for a given assembly, defined as the length N for which 50% of all bases in the sequences are in a sequence of length L < N) [4].

## Utility

### Web interfaces

**Figure 1:**
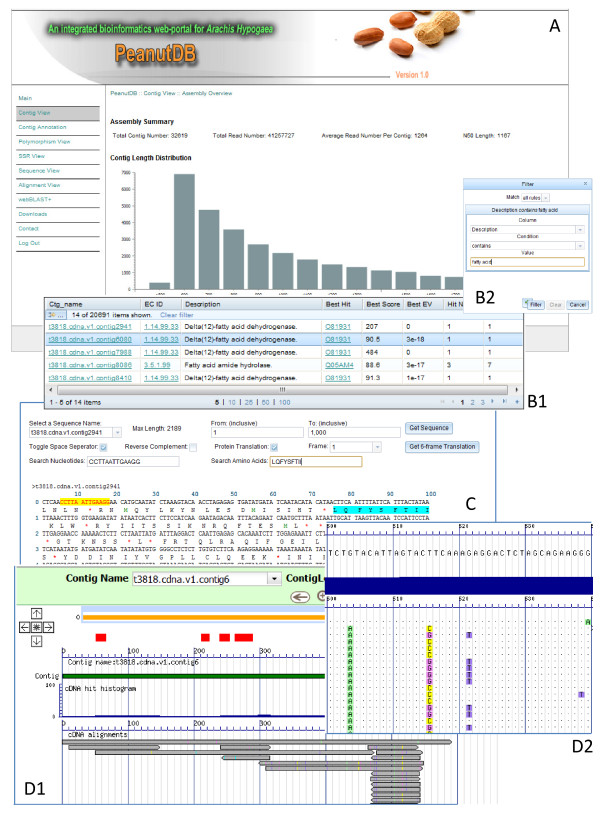
**The snapshots of PeanutDB web interfaces. Panel A**: the major web portal. **Panel B1**: The data grid view shows EC annotation within Contig Annotation. **Panel B2**: The data filter for data grid views. **Panel C**: the sequence viewer shows multiple functions for sequence manipulation. **Panel D1**: the alignment view in bird’s-eye resolution. **Panel D2**: the alignment view in nucleotide resolution that displays and highlights the differences between contig sequence and individual sequence reads.

As shown in Figure 1, PeanutDB web interfaces can be essentially categorized into three types: data grid views, sequence view and alignment view.

Data grid views present all data in an Excel sheet style and allow users to sort data in either ascending or descending order by clicking the column headers (Figure 1 B1). Moreover, they permit users to search and filter data by applying one single or up to 3 combined filtration conditions (Figure 1 B2). Concretely, data grid views include the following web interfaces. **Contig View** presents an overview of the whole transcriptome assembly (Assembly Overview), as well as individual contigs (Contig Summary), which contains the total number of sequence reads, contig length and coverage, and overall information about putative polymorphisms and SSRs detected for each contig. **Contig Annotation** allows users to navigate all available GO, EC and KEGG annotations for individual contigs, including annotation description, the best hit in UniProt (http://www.uniprot.org/) and the relevant significance (*i.e.*, the score and E-value of the best hit). For example, by applying the filtration condition of “*Description contains fatty acid*”, we can retrieve 14 (Figure 1 B1 and B2), 437 and 2,233 entries respectively for EC, KEGG and GO annotation that are related to fatty acid. **Polymorphism View** provides information for both the putative polymorphic positions along each contig (*e.g.*, the contig *t3818.cdna.v1.contig156* has A in the position 198 while 15 reads have G, 14 reads have C and 37 reads have A in this same position, http://bioinfolab.muohio.edu/txid3818v1/CBrowse/interface/ngctgbrowser.php?ctgname=t3818.cdna.v1.contig156&pos=198&zoom=2), and associated polymorphisms, either single-base SNPs and indels or multiple-base polymorphisms (*i.e.*, the contig *t3818.cdna.v1.contig156* has CTCG from position 471 to 474 while 28 reads have the same sequence CTCG and 3 reads have the 4-*nt* deletion here, http://bioinfolab.muohio.edu/txid3818v1/CBrowse/interface/ngctgbrowser.php?ctgname=t3818.cdna.v1.contig156&pos=471&zoom=2). **SSR View** describes detailed information for all SSRs detected for a given contig, including start and end position, repeat unit size, repeat number and identity (*i.e.*, 100% identity for perfect SSR).

Different from data grid views that adopt Excel sheet style for data representation, **Sequence View** empowers users by its highly interactive interface for examining and manipulating individual nucleotides of any given contig. Users can quickly extract the whole region or a sub-region of any contig sequence, and easily conduct reverse complementation, nucleotide counting and positioning, 6-frame translations, search and highlighting of nucleotide/amino acid motifs, and ORF finding (see Figure 1 C).

**Alignment View** offers both bird’s-eye and nucleotide-level resolutions of sequence alignments for each contig and its component sequence reads, as shown in Figure 1 D1 and D2. It provides an overview of the coverage profile along a contig, displays nucleotide differences (*i.e.*, mismatches, insertions and deletions) between any sequence read and contig sequence, and highlights detected SSRs and SNPs. **Alignment View** adopts Google map like technology so that all alignments can be viewed, dragged and moved smoothly. In addition, if the contig is very long, a user can easily navigate to a specific position by specifying the position in the text field “Site” and then clicking “Go” button or by dragging and moving the navigation scale bar. Clearly, it is a useful tool to examine assembly quality, genetic polymorphisms and/or sequencing errors closely.

### WebBLAST+

BLAST + [11] is a new suite of NCBI BLAST with many improvements in both performance and accuracy in comparison with the legacy BLAST. We have deployed this new tool in our website called webBLAST + so that a user can upload or copy-and-paste their sequences to do BLAST search against our peanut transcript assembly and NCBI peanut datasets including Core Nucleotides and GSS data. Up on the community requests, we will add more useful databases for blasting.

### Data download and release

PeanutDB is designed to be an open-access community database with high data transparency. All the data in abovementioned data grid views can be saved into client computers. We also provide a download page (http://bioinfolab.muohio.edu/txid3818v1/downloads.php) to enable bulk download. Our data release includes clean mRNA/cDNA sequence data, contig sequence data, contig annotation data, and among others.

## Discussions

Due to the low genetic diversity, complex genetic behaviors and large tetraploid genome, peanut remains to be one of less-studied crops with limited genomic resources. In recent years, however, more genomic resources are being generated for this non-model species [4]. With the next generation sequencing like 454 pyrosequencing and Illumina SBS, more and more transcriptome sequences are available for peanut. Furthermore, genome sequencing is just in its inception stage for this complex tetraploid crop, which quickly demands more cDNA/mRNA data for better genome annotation and gene prediction.

So far, the only public Peanut transcriptome database is NCBI Peanut UniGene Build#2 (http://www.ncbi.nlm.nih.gov/UniGene/UGOrg.cgi?TAXID=3818) which was developed on June 09, 2011. Using 37,621 mRNAs and 79,559 Sanger ESTs, this Build#2 contains 33,015 UniGene clusters, in which 17,256 are singletons with only one sequence read per contig. Unfortunately, this database has very basic web interfaces and limited data visualization. In contrast, our PeanutDB incorporates all mRNA, Sanger ESTs and 454 reads with much larger amount of Illumina reads recently generated by Institute of Plant Protection, Chinese Academy of Agricultural Science. Such data integration will definitely present a more comprehensive view of peanut transcriptome. Using both open-source and in-house bioinformatics tools, PeanutDB provides users with easy-to-use and seamlessly integrated web interfaces or views that facilitates data analysis and mining. For example, a user might want to find all contigs that are related to fatty acid metabolism, through the filtration in KEGG annotation view using “*Description contains fatty acid biosynthesis*”, he or she is able to retrieve 87 entries (79 contigs) that meet the criterion. Following the web links, the user can examine and manipulate the relevant contig sequence and visualize either the bird’s-eye view or nucleotide-level sequence alignment of the given contig. Moreover, using contig name, the user can retrieve all relevant simple sequence repeats and putative polymorphic positions, as well as polymorphism types (either single-base SNPs/indels vs. multiple-base mismatches/indels) and frequencies for a given contig.

Currently, *de novo* transcriptome assembly heavily utilizes sequence similarity. Sequence reads used for such assembly are often short/partial and only represent partial transcripts. Consequently, transcripts from homologous genes, including orthologs and paralogs, might have been clustered into the same contigs. On the other hand, in our current database release, we did not incorporate genotype information into contigs due to the fact that collecting genotype information for individual sequence reads and incorporating this information into the assembly is not an easy task, especially for Sanger ESTs submitted by many different labs and researchers. In our future database release, we will explore the effective way for such incorporation, so that biologists can use genotype information to validate true polymorphisms.

## Conclusion

With many easy-to-use and powerful web tools, PeanutDB provides the research community with a bioinformatics platform that will empower individual researchers in data integration, visualization and analysis/mining. Incorporating the most comprehensive datasets of peanut transcriptome sequences, our database is capable to present a new perspective about peanut genome dynamics. Using both open-source and our in-house tools, PeanutDB presents many seamlessly integrated web interfaces that allow users to search, filter, navigate and visualize easily the whole transcript assembly, its annotations, and detected polymorphisms and simple sequence repeats. In particular, our alignment view enables biologists to closely visualize and examine base-by-base the genetic polymorphisms, repeats and/or sequencing errors embedded in the transcriptome assembly. Without a doubt, PeanutDB will facilitate genomic research including molecular breeding in this less-studied crop species.

## Availability and requirements

The database website for PeanutDB is (http://bioinfolab.muohio.edu/txid3818v1). We have tested our web interfaces using Google Chrome, Mozilla FireFox (8.0 or above) and Microsoft Internet Explorer (9.0 or above). It is likely that our database works well with other internet browsers. For the best visualization effect and performance, we recommend Google Chrome and Mozilla FireFox. PeanutDB has an open access and all data can be downloaded through the website.

## Abbreviations

EST, Expressed Sequence Tag; SNP, Single Nucleotide Polymorphism; SSR, Simple Sequence Repeat.

## Competing interests

The authors declare that they have no competing interests.

## Authors’ contributions

CL and JZ managed and coordinated the whole project. XD, CS and JZ are responsible for peanut RNA sampling and Illumina sequencing. ES, PL, DL and LL carried out main database and web interface implementation. XD, ES, PL, DL and LL conducted extensive web testing. LC prepared the manuscript. All authors participated in manuscript writing and editing. All authors read and approved the final manuscript.

Xiaohong Duan and Emily Schmidt are co-first authors.
